# Multi‐Biomimetic Structural Colors Inspired by Pearls and Bird Feathers

**DOI:** 10.1002/adma.75074

**Published:** 2026-09-22

**Authors:** Zhiwei Wang, Qiuyu Cheng, Qiuyu Jin, Fu Tang, Wenbin Cao, Ayahisa Okawa, Takuya Hasegawa, Takehiro Goto, Natsumi Yamaguchi, Takumi Tanaka, Te‐Wei Chiu, Tohru Sekino, Shu Yin

**Affiliations:** ^1^ Institute of Multidisciplinary Research for Advanced Material (IMRAM) Tohoku University Sendai Japan; ^2^ School of Materials Science and Engineering University of Science and Technology Beijing Beijing China; ^3^ Research and Development Department Daito Kasei Kogyo CO. LTD. Osaka Japan; ^4^ Department of Materials and Mineral Resources Engineering National Taipei University of Technology Taipei Taiwan; ^5^ SANKEN The University of Osaka Osaka Japan; ^6^ Advanced Institute for Materials Research (WPI‐AIMR) Tohoku University Sendai Japan

**Keywords:** biomimetic materials, lamellar Ce_2_(CO_3_)_3_·8H_2_O, lamellar CeO_2_, non‐photonic crystal, structural color

## Abstract

One‐dimensional photonic crystals (1D PCs) enable precise control of light–matter interactions and display stable structural colors. However, their optical performance critically depends on strict periodicity and nanometer‐scale control of thickness, necessitating complex fabrication processes. In this work, we present a bio‐inspired structural coloration strategy that avoids these limitations by mimicking the structures of pearls and bird feathers. Specifically, a pearl‐like inorganic multilayer architecture lacking strict periodicity was synthesized via a facile solution‐based method. Distinct from traditional 1D PCs, this structure does not rely on precise periodicity, yet preserves the alternating refractive index modulation characteristic of 1D PCs. This disordered multilayer structure causes multiple reflections and partially coherent interference at internal interfaces, resulting in vivid and stable structural colors. Furthermore, inspired by the broadband‐absorbing melanin in bird feathers, surface‐integrated absorbing materials were introduced to selectively suppress non‐target interference. By tuning the absorbing materials, a continuous structural color spectrum covering the visible range can be achieved. The resulting biomimetic optical materials are readily processable and compatible with paints, plastics, coatings, and cosmetic applications.

## Graphical abstract

A dual‐biomimetic strategy mimicking pearls and bird feathers is proposed to construct full‐gamut structural colors. By integrating a non‐strictly periodic inorganic multilayer architecture with tunable light absorbers, this facile approach circumvents the complex fabrication of traditional 1D photonic crystals, offering immense potential for cosmetics, coatings, and plastics.

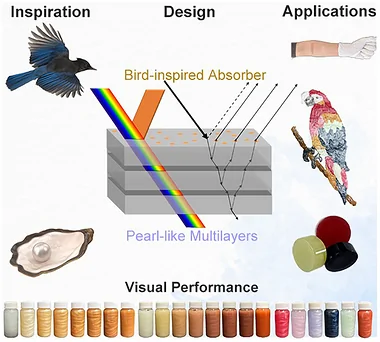

## 1 Introduction

Color, as a fundamental carrier of visual perception, arises from the interaction between light and matter [1, 2, 3, 4]. In nature, a rich diversity of nature structures, such as pearls, bird feathers, insect wings, and opals, display brilliant colors and distinctive optical effects [5, 6, 7, 8]. The mechanisms underlying these natural structural colors primarily originate from coherent and incoherent scattering within complex nanostructures and particles, leading to diffraction, interference, and polarization phenomena that collectively contribute to their optical appearance [9, 10]. With continuous advances in materials science, an integration between natural photonic structures and synthetic materials has been achieved [11]. Interference films, diffraction gratings, photonic crystals (PCs), and amorphous photonic crystals (APCs) observed in biological systems serve as important design principles for biomimetic structural color materials [3]. Among these, PCs and APCs have attracted extensive research attention in recent years.

One‐dimensional PCs (1D PCs) have attracted considerable attention owing to their simple structure and design flexibility. However, their color modulation relies on precise matching of refractive index contrast, layer thickness, and periodicity, resulting in stringent fabrication requirements [6, 12]. In addition, the structural colors of 1D PCs typically exhibit strong angle dependence, which limits their applicability in functional coatings requiring color stability. In contrast, APCs systems offer distinct advantages. Both pearls and the feathers of the Steller's jay (*Cyanocitta stelleri*) can be categorized as examples of APCs. Pearls are composed of lamellar structures lacking strict periodicity (Figure S1a,b), in which light undergoes multiple reflections and interference at the interlayer interfaces. The superposition of these different wavelengths collectively produces a broadband white reflection and endows the reflected light with a sense of depth and soft luster [13, 14]. Similar observations have been reported for biomimetic gyroid nanostructures, in which both crystalline and amorphous architectures can exhibit pronounced photonic responses despite differences in long‐range order [15, 16, 17, 18]. In the Steller's jay feathers, unlike in their albino counterparts, the selective absorption of the broadband optical background by melanin enhances color saturation, leading to more vivid structural color (Figure S1c–f) [19, 20, 21]. Therefore, integrating the layered interference characteristics of pearls with the selective absorption mechanism inspired by Steller's jay feathers provides a promising strategy for constructing novel biomimetic optical materials that simultaneously exhibit vivid structural coloration and multidimensional visual effects.

For the construction of this biomimetic optical system, Ce_2_(CO_3_)_3_·8H_2_O was selected as a particularly suitable matrix material. Its anisotropic crystal growth favors the formation of plate‐like crystals [22], while the interlayer water incorporated within its crystal structure is considered to contribute to the development of the multilayer morphology [23]. Consequently, the multilayer structure can be generated directly during crystal growth without requiring an additional assembly process, thereby considerably simplifying material fabrication. Upon calcination, Ce_2_(CO_3_)_3_·8H_2_O is converted into chemically and thermally stable CeO_2_ while largely retaining its original multilayer morphological features. FeOOH was selected as the light‐absorbing component because of its pronounced absorption in the short‐wavelength region of the visible spectrum and its good compatibility with the cerium‐based matrix. Moreover, subsequent calcination converts FeOOH into stable and low‐toxicity Fe_2_O_3_, further improving the structural and chemical stability of the biomimetic optical materials.

In this study, the optical mechanisms of two natural amorphous structures were integrated into an inorganic system to construct composite layered structures that generate bright, dynamic, and visually layered structural colors. Inspired by the multilayered structure of pearls, layered cerium carbonate and cerium oxide were synthesized via preferential orientation, producing samples with a dynamic white pearlescent appearance. By depositing light‐absorbing materials on the surface, part of the visible interference light in broadband reflection and scattering was selectively absorbed, leading to complementary coloration while preserving the dynamic and layered visual effects inherent to structural colors. In contrast to conventional layered structural color materials, these novel biomimetic optical materials generate dynamic interference colors without stringent control of layer thickness, and offer facile synthesis.

## 2 Results and Discussion

### 2.1 Color Generation Mechanism and Biomimetic Structural Synthesis

Inspired by natural structures such as pearls and bird feathers, this study presents a strategy for designing tunable structural colors by integrating multiple nature‐derived structural features. In pearls, the lamellar structure induces multiple interfacial reflections and partially coherent interference, leading to broadband reflection. The superposition and mixing of different wavelengths result in a soft, bright white pearlescence at the macroscopic scale [14, 24]. Meanwhile, the feathers of the Steller's jay contain absorbing materials that efficiently suppress wavelengths unrelated to the target structural coloration, thereby enhancing coherent reflection at specific wavelengths and producing a vivid and pure blue hue [19, 20, 21]. To mimic such natural non‐iridescent structural colors, researchers have attempted to introduce light‐absorbing materials into APCs as the structural components [25, 26] or underlying background [20, 27, 28]. These bio‐inspired structural color materials have been explored for various applications [29, 30, 31]. In this work, a multi‐biomimetic optical material was developed based on the synergistic coloration mechanisms of these two natural structures (Figure 1a). Inspired by the lamellar structure of pearls, a multilayered material was designed and synthesized. Similar to natural pearls, this material features alternating refractive indices and a distribution of layer thicknesses lacking strict periodicity, giving rise to interference through multiple interfacial reflections and resulting in soft, broadband structural color reflection. Furthermore, surface‐deposited absorbing materials selectively suppress broadband background reflection, thereby enhancing the perceptual contrast of the remaining structural reflection. The resulting material exhibits vivid colors with a characteristic pearlescent luster (Figure 1b). In our system, the strategy is fundamentally different from simply suppressing an incoherent scattering background. The non‐periodic multilayer architecture generates multiple interference‐related reflection features across the visible region, whose superposition produces a broadband white reflection. The introduced absorbing components then selectively attenuate the nontarget portions of these interference‐related reflections, increasing the relative contribution of the desired wavelength region and thereby enhancing the color saturation (all absorber types and concentrations are presented in Tables S1–S4). The observed coloration results from the combined contributions of micro‐/nanostructure‐induced optical effects and pigment absorption. Since the structural optical response remains the dominant color‐generation mechanism, this hybrid coloration is still generally classified as structural color [32].

**FIGURE 1:**
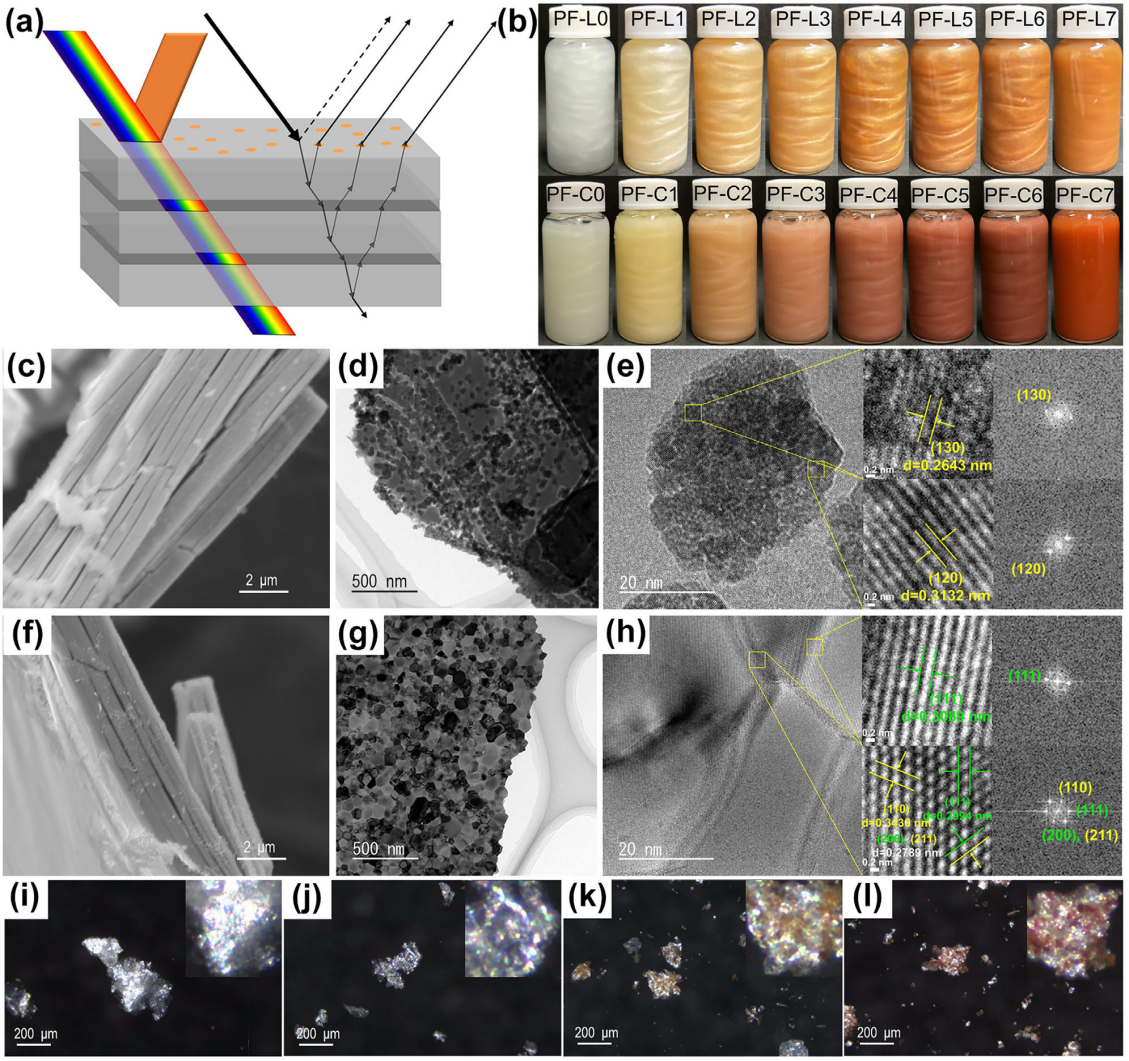
Dual‐biomimetic strategy for structural color modulation. (a) Schematic diagram of the dual‐biomimetic structural color mechanism. (b) Dynamic images of the samples in water before and after sintering. (c) Side‐view SEM image of Sample PF‐L0. (d) TEM image of Sample PF‐L5 showing surface‐loaded absorbing materials. (e) HR‐TEM image with the corresponding FFT pattern of PF‐L5's absorbing materials (FeOOH) on the substrate, with FeOOH lattice planes highlighted in yellow. (f) Side‐view SEM image of Sample PF‐C0. (g) TEM image of Sample PF‐C5. (h) HR‐TEM image with the corresponding FFT pattern of the Sample PF‐C5, where the CeO_2_ lattice planes are marked in green and the Fe_2_O_3_ lattice planes are highlighted in yellow. (i–l) Optical microscope images of interference phenomena (i) PF‐L0, (j) PF‐C0, (k) PF‐L5, and (l) PF‐C5.

Based on this concept, Ce_2_(CO_3_)_3_·8H_2_O, which exhibits preferred orientation and tendency to form lamellar structures, was selected as the building unit to mimic the pearl‐like structure. A liquid‐phase synthesis strategy was employed to construct an amorphous photonic crystal (APC) structure comprising alternating thick Ce_2_(CO_3_)_3_·8H_2_O layers and thin air gaps (Figure 1c). The significant refractive index contrast between Ce_2_(CO_3_)_3_·8H_2_O and air caused multiple interfacial reflections and coherent interference [33]. To investigate the feasibility of the proposed optical design, transfer matrix method (TMM) simulations were performed using a non‐periodic multilayer model composed of alternating Ce_2_(CO_3_)_3_·8H_2_O and air layers [34, 35]. The physical thicknesses of the inorganic layers and interlayer air gaps were extracted from the cross‐sectional SEM image in Figure 1c and incorporated into the model to reproduce the structural disorder observed experimentally. The simulated spectrum exhibits multiple reflection features distributed throughout the visible wavelength range (Figure S2a), and the spectral overlap of these features gives rise to an overall broadband white reflection. Furthermore, with increasing angle of incidence, the simulated reflection features shift toward shorter wavelengths (Figure S2a), further supporting the contribution of multilayer interference to the broadband optical response [36]. Subsequently, iron‐containing compounds were deposited onto the surface of the Ce_2_(CO_3_)_3_·8H_2_O multilayer structure via self‐assembly. These samples are denoted as PF‐L and further labeled as PF‐L0–PF‐L7 based on the loading amount of the absorbing material (Tables S1, S5, and S6; Note S1 and Figure S3). Transmission electron microscopy (TEM) and high‐resolution transmission electron microscopy (HR‐TEM) reveal the successful deposition of the absorbing materials as nanoscale clusters on the substrate surface (Figure 1d,e). Corresponding fast Fourier transform (FFT) analysis confirms that the surface iron‐containing species is FeOOH, based on the matching of measured interplanar spacings with standard crystallographic data (Figure 1e). Given the poor physicochemical stability of carbonate hydrates, the samples were further subjected to calcination at 800°C, with the calcination temperature was determined based on TG–DTA analysis (Figure S4). The calcined samples (denoted as PF‐C, PF‐C0–PF‐C7 (Table S1)) retained their plate‐like multilayered structural features (Figure 1f). However, the originally smooth surface evolved into a roughened morphology composed of interconnected nanoparticles (Figure 1g). HR‐TEM and the corresponding FFT analyses (Figure 1h) confirm that this calcination process transformed the Ce_2_(CO_3_)_3_·8H_2_O into structurally stable CeO_2_, while the absorbing material FeOOH was converted into Fe_2_O_3_. Notably, the resulting Fe_2_O_3_ exists in an embedded form within the surface CeO_2_ matrix. Owing to these bio‐inspired structural features, the optical materials exhibit multiple interference‐related colors under optical microscopy (Figure 1i,j), and the superposition of these local reflections gives rise to an overall white‐light reflection. These multicolored spots are consistent with the spectral features obtained from the TMM simulations (Figure S2a), suggesting that different local multilayer configurations generate interference‐related reflections at different wavelengths [37], which collectively produce the broadband white reflection observed at the macroscopic scale. After the introduction of absorbing materials, nontarget interference contributions are effectively suppressed, leading to clear and distinguishable color variations (Figure 1k,l). Consequently, compared with conventional absorption‐dominated coloration, the resulting samples display interference‐based structural colors with enhanced visual depth and pronounced hierarchical contrast (Figure 1b; Movies S1 and S2).

The similar optical characteristics observed before and after calcination are closely associated with the retention of the disordered multilayer architecture. To quantitatively verify this structural disorder, cross‐sectional SEM images were collected from multiple particles and different local regions (Figure S5), and the thicknesses of the inorganic layers and interlayer air gaps were statistically analyzed. The resulting thickness distributions confirmed the non‐uniform and disordered nature of the synthesized multilayer substrate (Note S2). Although calcination induced phase transformation and surface roughening, the overall multilayer configuration and its disordered characteristics were retained. To further investigate the influence of structural disorder on the optical response, TMM simulations were performed using the experimentally measured multilayer configurations of representative particles. Owing to differences in the local layer‐thickness distributions, the simulated spectral profiles varied among the particles. Nevertheless, all simulated spectra exhibited multiple interference‐related reflection features distributed across the visible region (Figure S2b). The overlap of these reflection features is considered to be the primary origin of the broadband white reflection. Since the disordered multilayer architecture was preserved after calcination, the corresponding broadband interference‐related optical characteristics were also retained.

### 2.2 Structural Characterizations of Multi‐Biomimetic Optical Materials

Figure 2a,b presents the x‐ray diffraction (XRD) patterns of the samples exhibiting structural coloration before and after calcination, respectively. The crystal structure of the Ce_2_(CO_3_)_3_·8H_2_O substrate was analyzed via the Rietveld refinement method. The refined XRD spectra with satisfied fitting (Figure 2c; Tables S7 and S8) indicate that the obtained Ce_2_(CO_3_)_3_·8H_2_O substrate has a typical layered structure (space group: Pmn2_1_; *a* = 9.52 Å, *b* = 8.46 Å, *c* = 8.92 Å, *α* = 90°, *β* = 90°, and *γ* = 90°). The lattice water molecules are intercalated and distributed between the layers (Figure S6,). Further refinement results reveal that Ce_2_(CO_3_)_3_·8H_2_O exhibits a pronounced preferential orientation along the (010) plane during crystal growth (Figure 2c). This anisotropic growth behavior promotes the formation of uniform‐sized multilayer sheets composed of stacked plate‐like building units (Figures 1c and 2d,e; Figure S7a,c). In this multilayer structure, the variation in layer thickness among Ce_2_(CO_3_)_3_·8H_2_O layers leads to interference of reflected light at different wavelengths. As a result of the superposition and mixing of these wavelengths, the multilayer cerium carbonate structure exhibits a soft, pearl‐like white luster [14, 24] The SAED pattern exhibits discrete diffraction spots, indicating a single‐crystalline nature. Ce_2_(CO_3_)_3_·8H_2_O; however, is unstable under high‐energy electron‐beam irradiation. With prolonged exposure, the SAED patterns gradually evolve from discrete diffraction spots to ring‐like features (Figure 2f and Figure S8). Comparison with XRD refinement results shows that the interplanar spacings derived from SAED are slightly smaller. This discrepancy is associated with dehydration induced by exposure to high‐energy electron‐beam irradiation, which leads to lattice contraction along the *b*‐axis.

**FIGURE 2:**
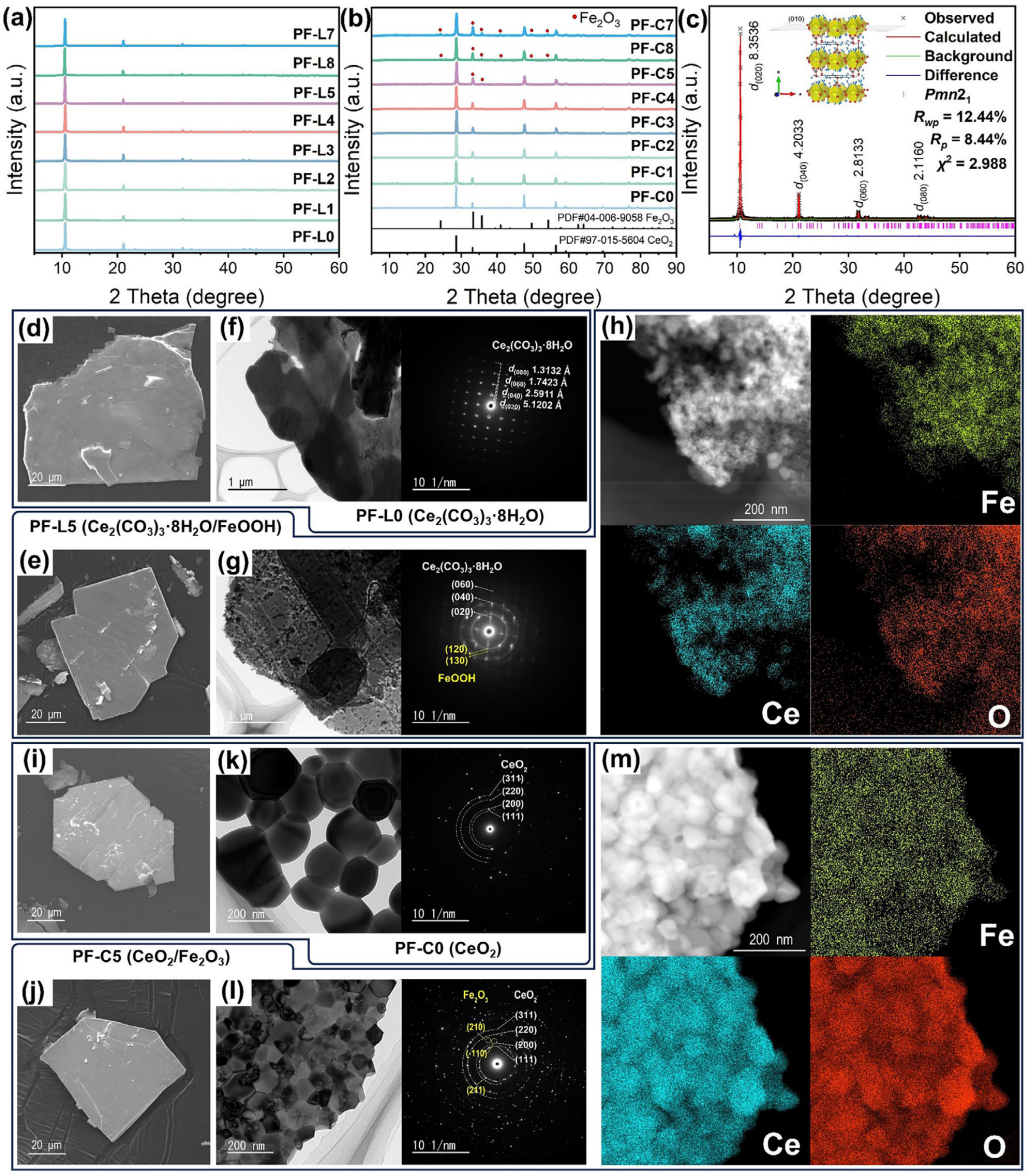
Structural characterizations of multi‐biomimetic optical materials. XRD patterns of the as‐prepared materials based on (a) Ce_2_(CO_3_)_3_·8H_2_O and (b) CeO_2_ substrates. (c) XRD refinement and structure images of PF‐L0. SEM images of (d) PF‐L0 and (e) PF‐L5. TEM images and corresponding SAED patterns of (f) PF‐L0 and (g) PF‐L5. (h) STEM image and the corresponding EDX elemental mapping images of PF‐L5. SEM images of (i) PF‐C0, and (j) PF‐C5. TEM images and corresponding SAED patterns of (k) PF‐C0, and (l) PF‐C5. (m) STEM image and the corresponding EDX elemental mapping images of PF‐C5.

After the introduction of the absorbing materials, no diffraction peaks attributable to these species are observed (Figure 2a). This is mainly attributed to the low crystallinity of the introduced FeOOH (Figure S9), which renders its diffraction signals are difficult to resolve in the XRD patterns. TEM and energy dispersive x‐ray spectroscopy (EDX) observations show that these low‐crystallinity FeOOH species are evenly distributed on the sample surface as nanoparticles (Figure 2g,h; Figures S10 and S11). These nanoscale absorbers selectively absorb the reflected interference light from the multilayer substrate, enabling effective control of the resulting structural coloration. To obtain biomimetic optical materials with improved stability, the samples were subjected to calcination. After calcination, FeOOH was converted into Fe_2_O_3_ with higher crystallinity, and the corresponding characteristic diffraction peaks became discernible (Figure 2b). Meanwhile, the substrate was transformed from Ce_2_(CO_3_)_3_·8H_2_O into CeO_2_. Notably, the overall structural characteristics were well preserved, evolving into a uniform plate‐like structure composed of alternately stacked CeO_2_ layers and air gaps (Figures 1f and 2i,j; Figure S7c,d). The retention of this multilayered structure ensures that the overall optical appearance, characterized by pronounced visual depth and hierarchical effects, remains largely intact (Figure 1b). TEM observations performed in relatively thin regions reveal that the original plate‐like single‐crystalline structure transforms into a plate‐like polycrystalline structure composed of nanosized particles (Figure 2k,l). This result indicates that the sintering process induces a crystal‐structure transition from single‐crystalline to polycrystalline. Notably, the introduction of iron‐containing absorbing materials significantly affects the size of these nanoparticles. Compared with the pristine samples, the nanoparticles formed after incorporation of the absorbing materials are significantly smaller (Figure 2k,l). This illustrates the ability of a dispersed secondary phase to prevent particle coarsening during thermal processing [38]. Further SEM observations indicate that this size reduction predominantly occurs in the surface region of the plate‐like structures (Figure S12). The presence of these fine nanoparticles increases surface roughness, which weakens specular reflection and leads to a noticeable reduction in the flickering effect (Figure 1b). EDX analysis further confirms that the resulting Fe_2_O_3_ is uniformly distributed across the sample surface (Figure 2m; Figures S13 and S14). Upon further increasing the calcination temperature, the samples were transformed into plate‐like particles composed of interconnected nanoparticles (Figure S15a–c), accompanied by the loss of the characteristic multilayer architecture. Although the crystalline phase remained unchanged (Figure S15d), the interference‐related optical features were no longer observed (Figure S15e,f). This structural‐optical correlation further supports the important contribution of the multilayer architecture to the interference‐related optical response.

### 2.3 Characterization and Analysis of Optical Properties

The oxidation states and the crystal field environments can profoundly affect the electronic transitions of transition‐metal and rare‐earth ions, thereby determining their absorption behavior in the visible region. Therefore, identifying the specific absorption peaks of the introduced absorbing materials in the visible range and excluding the intrinsic absorption of the layered substrate are crucial for elucidating the structural color evolution mechanism in this system. To this end, x‐ray photoelectron spectroscopy (XPS) and UV–vis–NIR spectroscopy were employed to characterize the biomimetic optical materials before and after calcination.

Cerium in the Ce_2_(CO_3_)_3_·8H_2_O substrate exists predominantly in the +3 oxidation state (Figure 3a). After the introduction of iron‐containing compounds, a small amount of Ce^4+^ was observed, which is likely related to surface oxidation induced by prolonged exposure to air during the assembly process (Figure 3a and Figure S16) [39, 40]. The samples also exhibited characteristic Fe^3+^ Gupta and Sen (GS) multiplets (Figure 3b), arising from the crystal‐field splitting of high‐spin Fe^3+^ (3d^5^) with unpaired d electrons [41, 42]. According to UV–vis–NIR spectral analysis (Figure 3c), the absorption of Ce^3+^ mainly occurs in the ultraviolet region. Consequently, the PF‐L0 sample exhibits negligible absorption in the visible range, displaying white interference coloration induced by the multilayer structure (Figures 1b,i and 3c). Based on the crystal structure of the FeOOH component, Fe^3+^ occupies octahedral crystal‐field environments (Figure S17). Accordingly, analysis of the UV–vis–NIR spectra in conjunction with the Tanabe–Sugano diagram indicates that the characteristic absorptions of Fe^3+^ in the visible region originate from charge‐transfer transitions and *d–d* transitions (Figure 3c and Figure S18) [43, 44, 45]. These iron‐based absorbers introduce an absorption band in the blue region, selectively attenuating the blue interference light reflected from the substrate and yielding an orange‐yellow appearance. As the concentration of the absorber increases, blue‐light absorption becomes more pronounced. Meanwhile, the red component gradually dominates, and the overall brightness decreases (Figure 3d).

**FIGURE 3:**
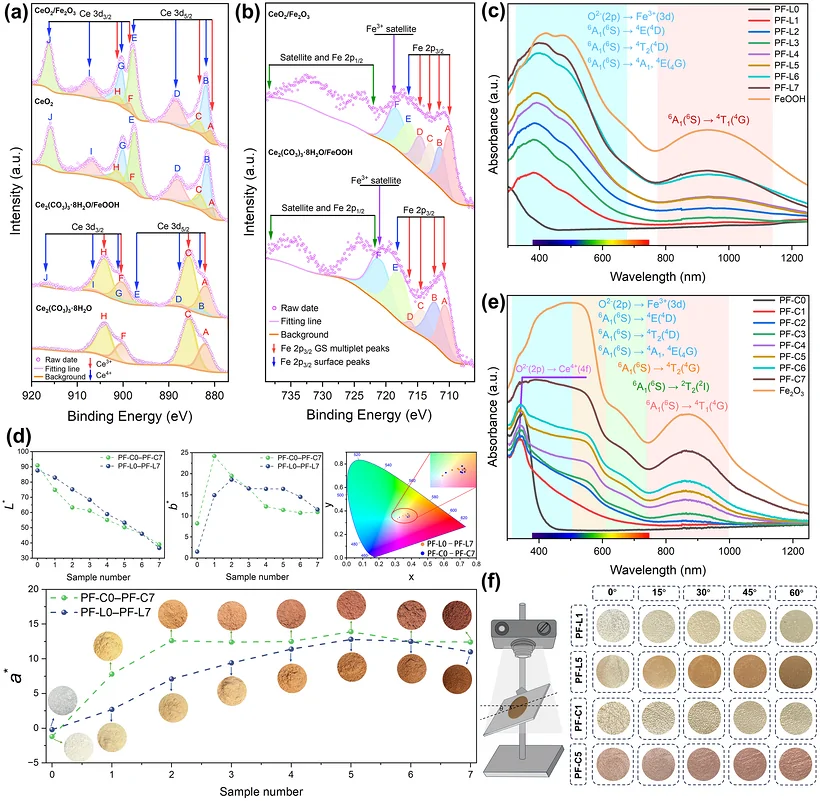
Chemical state and optical characterization of multi‐biomimetic materials. (a) High‐resolution Ce 3d XPS spectra of Ce_2_(CO_3_)_3_·8H_2_O (PF‐L0), Ce_2_(CO_3_)_3_·8H_2_O/FeOOH (PF‐L5), CeO_2_ (PF‐C0), and CeO_2_/Fe_2_O_3_ (PF‐C5). (b) High‐resolution Fe 2P XPS spectra of Ce_2_(CO_3_)_3_·8H_2_O/FeOOH (PF‐L5) and CeO_2_/Fe_2_O_3_ (PF‐C5). (c) UV–vis–NIR absorption spectra of as‐prepared Ce_2_(CO_3_)_3_·8H_2_O‐based materials with different Fe loadings. (d) CIE L^*^a^*^b^*^ color coordinates with photographs and CIE 1931 chromaticity coordinates of multi‐biomimetic materials. Here, L^*^ denotes lightness, a^*^ denotes the green (−) to red (+) axis, and b^*^ denotes the blue (−) to yellow (+) axis. (e) UV–vis–NIR absorption spectra of as‐prepared CeO_2_‐based materials with different Fe loadings. (f) Digital photographs of multi‐biomimetic materials at different incident angles.

After calcination, the XPS spectra indicate that Ce^3+^ was not fully oxidized (Figure 3a and Figure S16) [39, 40, 46]. The residual Ce^3+^ induces a slight redshift of the Ce^4+^ band gap, extending its absorption tail into the blue region of the visible spectrum and resulting in weak blue‐light absorption (Figure 3e) [42] As shown in Figure 1b, the PF‐C0 sample exhibits a faint yellowish appearance. Concurrently, the absorbing materials undergo a phase transformation during calcination, converting from FeOOH to Fe_2_O_3_. Notably, despite this phase transition, both FeOOH and Fe_2_O_3_ exhibit pronounced visible‐light absorption, thereby maintaining stable structural coloration. For the Fe_2_O_3_ formed after calcination, the XPS spectra show the characteristic features of Fe^3+^ exclusively, confirming that the oxidation state of iron remains stable at +3 (Figure 3a). In addition, Fe^3+^ within the Fe_2_O_3_ lattice also occupies the octahedral crystal field environment (Figure S17), resulting in spectral characteristics that are largely consistent with samples PF‐L1–PF‐L7. However, subtle variations in the chemical environment and local structure still lead to differences in the crystal‐field environment of Fe^3+^. These variations introduce a weak absorption band in the yellow‐green region of the visible spectrum, which can be attributed to spin‐forbidden *d–d* transitions, ultimately giving rise to discernible differences in the observed coloration (Figure 3d) [43, 44, 45]. These differences are clearly observed under optical microscopy. Samples without absorbing materials reflect light across a broad range of visible wavelengths and appear nearly white (Figure 1i,j). After the introduction of the absorbers, reflection in the blue region is significantly suppressed, whereas the red component is relatively enhanced and effectively reflected (Figure 1k,l). In addition, owing to differences in absorption characteristics, samples loaded with FeOOH partially retain and reflect light in the green and yellow regions (Figure 1k). These color variations are also clearly visible in digital photographs, further confirming that the effectiveness of structural color can be adjusted by selecting absorbing materials (Figure 1b). Furthermore, as shown in Figure 1b, when the amount of absorber content exceeds a certain threshold, the interference effect from the layered structure is completely masked, resulting in a gradual shift from coloration dominated by structural color to absorption dominance, along with the disappearance of dynamic optical effects. Beyond changes in optical mechanisms, distinct macroscopic color differences are also evident. The samples PF‐L1–PF‐L7 change from a golden‐apricot hue to pumpkin orange, while the samples PF‐C1–PF‐C7 change from brownish red to orange–red. Variations in color difference and chromaticity parameters further corroborate the sensitive influence of subtle crystal‐field differences on material coloration. And they also reveal the perceptible color changes associated with the transition from structural to absorption coloration (Figure 3d).

The biomimetic optical materials constructed in this work exhibit structural colors with noticeable depth and hierarchical visual effects. Digital photographs and multi‐angle reflectance spectra indicate that change in the angle primarily affects the gloss and reflectance intensity of the samples, while no obvious macroscopic hue shift is detected within the measured angular range. The decrease in reflectance intensity with increasing angle is consistent with the overall angular trend predicted by the TMM simulations (Figure 3f and Figures S19–S21). As shown in the CIE chromaticity diagram (Figure 3d and Figure S22a–d), the color distribution is concentrated in the complementary‐color region because of the selective visible‐light absorption by the absorbing materials. This indicates that a single type of light‐absorbing component limits the achievable color range of such biomimetic optical systems. To achieve full‐gamut structural color tuning and further validate the generality and scalability of the dual‐biomimetic mechanism, multiple absorbing materials with distinct absorption characteristics were subsequently incorporated into the system.

### 2.4 General Applicability of Dual‐Biomimetic Mechanisms for Structural Color Construction

Once light‐absorbing agents with specific absorption characteristics are introduced and immobilized onto the substrate surface, they selectively absorb different interference components generated by multiple internal reflections within the substrate, thereby giving rise to distinct spectral features (Figure 4a and Figures S22b,e–j). Notably, these components are exclusively deposited on the substrate surface without forming any new phases (Figure S23). The selection and assembly involved in this biomimetic structural color mechanism exhibit excellent generality with respect to the choice of absorptive components, as both inorganic and organic materials can be effectively incorporated into the system. By tuning the type and concentration of absorbing materials, continuous spectral coverage across the visible region can be readily achieved (Figure 4c,d and Figures S24–S29). In contrast to 1D PCs with similar layered structures, the structural color system constructed via this dual‐biomimetic strategy does not rely on strictly periodic stacking or precise control of individual layer thicknesses to obtain the desired coloration [11, 47, 48, 49]. In other words, this color‐generation mechanism avoids complex and high‐precision fabrication processes. Instead, its color modulation is primarily governed by the type and loading amount of the incorporated absorbers. Through the appropriate selection of absorptive components alone, interference‐based structural colors with dynamic visual effects and pronounced depth can be realized (Figure 4a and Figures S24–S29; Movie S3). Consistent with earlier observations, these samples show angle‐dependent behavior that manifests mainly as changes in gloss rather than hue (Figures S30–S35).

**FIGURE 4:**
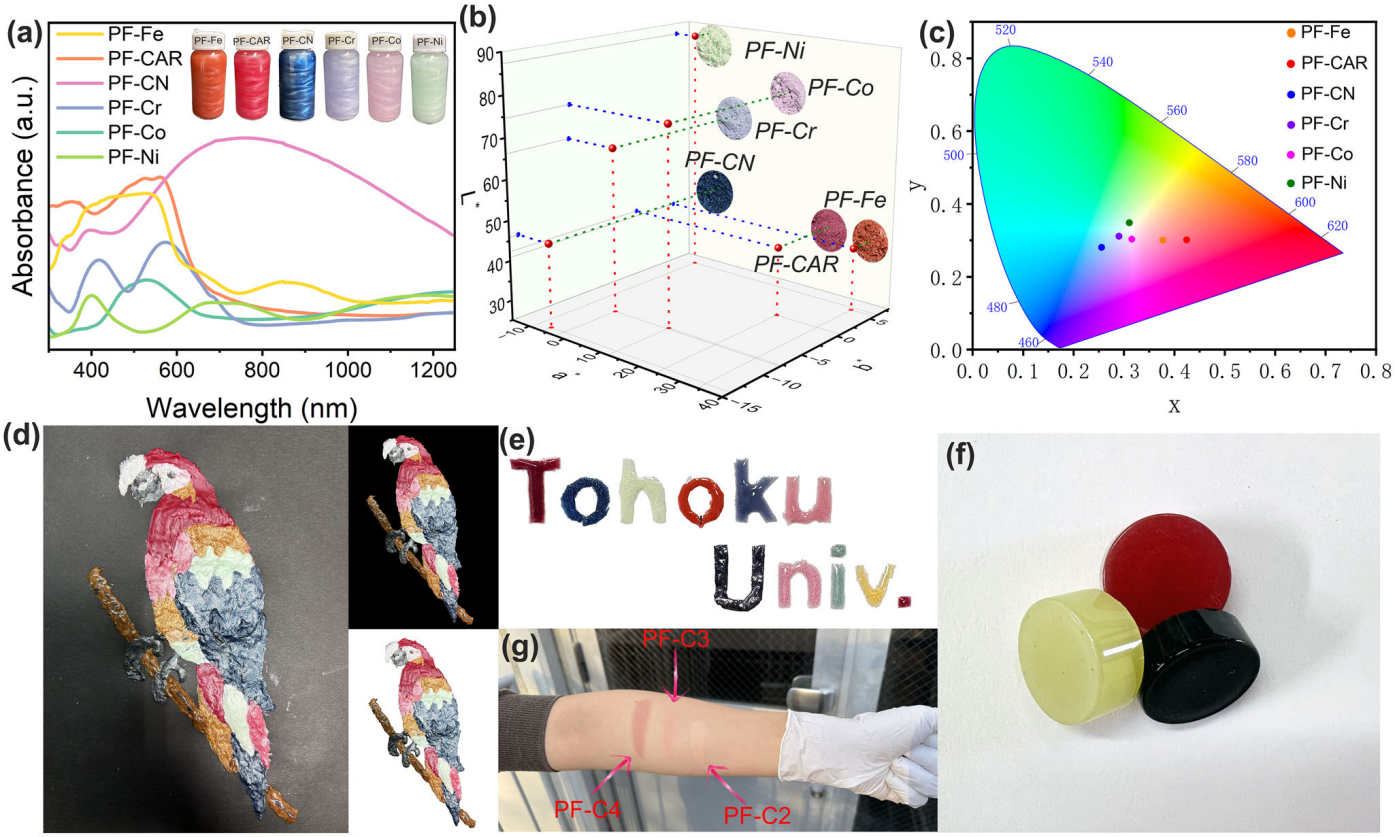
General applicability of structural color construction via a dual‐biomimetic mechanism. (a) UV–vis–NIR absorption spectra of as‐prepared Ce_2_(CO_3_)_3_·8H_2_O‐based materials loaded with different absorbing materials, along with dynamic images of the samples dispersed in water. (b) CIE L^*^a^*^b^*^ color coordinates and corresponding photographs of the multi‐biomimetic materials. (c) CIE 1931 chromaticity diagram of the multi‐biomimetic materials. (Surface deposition of different absorbing materials. PF‐Fe: Fe_2_O_3_; PF‐CAR: Carmine; PF‐CN: Fe_4_[Fe(CN)_6_]_3_; PF‐Cr: Cr‐containing compound; PF‐Co: Co‐containing compound; PF‐Ni: Ni‐containing compound) (d) A colorful parrot pattern painted on black cardstock using a simple mixture of the Bioinspired structural color materials and water. (e) The simplest coating formulation prepared by mixing the Bioinspired structural color materials with glycerol (serving as the binder), used to write “Tohoku Univ.” on cardstock. (f) Photograph of PMMA composites incorporating the multi‐biomimetic materials. (g) Photographs of CeO_2_/Fe_2_O_3_‐based multi‐biomimetic materials with different amounts of absorbing materials applied onto human skin.

The multi‐biomimetic optical materials exhibit an exceptionally broad color gamut, excellent temporal and chemical stability, and high reproducibility, highlighting their considerable potential for diverse applications. To further illustrate their prospects, we systematically explored their applicability in coatings, plastics, and cosmetic formulations. Using only the multi‐biomimetic optical materials, we created a parrot image containing more than 10 distinct color tones (Figure 4d), visually highlighting the richness of the achievable coloration. When mixed with glycerol to form a simplified paint system, the material was used to successfully inscribe the letters ‘Tohoku Univ.’ (Figure 4e). Furthermore, the materials were incorporated into the PMMA matrix and subjected to thermal curing to obtain uniformly colored PMMA bulk samples (Figure 4f), indicating excellent dispersion and color stability. Experimental results show that the color gamut of samples PF‐C2–PF‐C4 substantially overlaps with the human skin‐tone space, enabling skin‐tone matching while simultaneously providing brightening and coverage effects (Figure 4g; Movie S4). Meanwhile, the fabrication process also exhibits excellent scalability, further supporting its potential for practical applications. These results demonstrate the preliminary feasibility of incorporating the present biomimetic optical materials into cosmetic formulations while retaining their characteristic optical appearance after application to the skin. Cerium oxide has previously been investigated and used as a functional or light‐scattering component in cosmetic‐related formulations [50, 51], while iron oxides have an established history of use as cosmetic colorants under applicable regulatory specifications and conditions. These precedents offer a practical basis for exploring the CeO_2_/Fe_2_O_3_ system in cosmetic optical applications, further reinforcing the feasibility and potential of this material platform for aesthetic and practical uses.

## 3 Conclusion

In summary, we proposed a facile and scalable strategy to construct multi‐biomimetic structural color optical materials by integrating the layered structure of the pearl with the absorption characteristics of Steller's jay feathers. Taking advantage of the preferential orientation growth of Ce_2_(CO_3_)_3_·8H_2_O along the (010) plane, a pearl‐like layered structure was successfully constructed, establishing a previously unexplored structural‐color platform. Owing to the thickness variation within the multilayered structure, irregular multilayer interference is generated, enabling the superposition of different wavelengths of light and resulting in a bright white structural background. The subsequent assembly of light‐absorbing materials onto the surface introduces selective absorption, thereby producing vivid structural colors. By tuning the absorption characteristics and loading amount of the absorbers, both the color hue and its visibility can be precisely regulated. Furthermore, thermal calcination can be applied according to material requirements to obtain more stable inorganic structures. Notably, although the material shares structural similarities and optical principles with 1D PCs, an integrated multilayer reflector is intrinsically formed without requiring separate self‐assembly or layer‐by‐layer fabrication, while the resulting structural color is not strictly constrained by periodicity or precise layer thickness. This feature provides exceptional tolerance in fabrication and expands design flexibility. The developed artificial structural colors exhibit promising potential for applications in coatings, cosmetics, plastics, and other optical functional materials. The study of these dual‐biomimetic optical materials not only opens a new avenue for structural color research, but also broadens the strategies available for artificially reproducing natural optical systems.

## Author Contributions

**Zhiwei Wang**: conceptualization, investigation, methodology, formal analysis, data curation, Writing – original draft. **Qiuyu Cheng**: investigation, writing – review and editing, methodology, visualization, validation, formal analysis. **Qiuyu Jin**: investigation, writing – review and editing, visualization. **Fu Tang**: investigation, writing – review and editing, methodology. **Wenbin Cao**: methodology, investigation, writing – review and editing. **Ayahisa Okawa**: investigation, writing – review and editing. **Takuya Hasegawa**: investigation, writing – review and editing. **Takehiro Goto**: investigation, writing – review and editing, validation. **Natsumi Yamaguchi**: investigation, writing – review and editing, validation. **Takumi Tanaka**: investigation, writing – review and editing, validation. **Te‐Wei Chiu**: investigation, writing – review and editing. **Tohru Sekino**: writing – review and editing, investigation, resources. **Shu Yin**: conceptualization, investigation, funding acquisition, writing – review and editing, methodology, supervision, project administration, resources, software.

## Ethics Statement

CeO_2_/Fe_2_O_3_‐based multi‐biomimetic materials with different amounts of absorbing materials applied onto human skin were applied to human skin on a co‐author participant's arm (Figure 4(g)). The experiment involving the application of the material to human skin was performed by one of the authors, Qiuyu Cheng, in strict accordance with the relevant ethical guidelines and applicable regulations. Written informed consent was obtained from the participant prior to the experiment.

## Conflicts of Interest

The authors declare no conflicts of interest.

## Supporting information




**Supporting File 1**: adma75074‐sup‐0001‐SuppMat.docx.

**Supporting File 2**: adma75074‐sup‐0002‐MovieS1.mp4.

**Supporting File 3**: adma75074‐sup‐0003‐MovieS2.mp4.

**Supporting File 4**: adma75074‐sup‐0004‐MovieS3.mp4.

**Supporting File 5**: adma75074‐sup‐0005‐MovieS4.mp4.

## Data Availability

The data that support the findings of this study are available from the corresponding author upon reasonable request.
