# Supplementary material for: Multi‐Biomimetic Structural Colors Inspired by Pearls and Bird Feathers
Source: Adv Mater. 2026 Sep 22;38(62):e75074. doi: 10.1002/adma.75074 (PMC13741117; doi:10.1002/adma.75074)
Supplement: Supplementary file 1 [file ADMA-38-e75074-s002.docx]

Supporting Information

**Multi-Biomimetic Structural Colors Inspired by Pearls and Bird Feathers**

Zhiwei Wang^1^, Qiuyu Cheng^1^, Qiuyu Jin^1^, Fu Tang^1, 2^, Wenbin Cao^2^, Ayahisa Okawa^1^, Takuya Hasegawa^1^, Takehiro Goto^3^, Natsumi Yamaguchi^3^, Takumi Tanaka^3^, Te-Wei Chiu^4^, Tohru Sekino^5^, Shu Yin^1, 6, *^

^1^Institute of Multidisciplinary Research for Advanced Material (IMRAM), Tohoku University, 2-1-1 Katahira, Aoba-ku, Sendai, 980-8577, Japan

^2^School of Materials Science and Engineering, University of Science and Technology Beijing, Beijing 100083, China

^3^Research and Development Department, Daito Kasei Kogyo CO., LTD., 1-6-28 Akagawa, Asahi-ku, Osaka 535-0005 Japan

^4^Department of Materials and Mineral Resources, Engineering, National Taipei University of Technology, No. 1, Section 3, Chung-Hsiao East Road, Taipei 106, Taiwan

^5^SANKEN, The University of Osaka, Mihogaoka 8-1, Ibaraki, Osaka 567-0047, Japan

^6^Advanced Institute for Materials Research (WPI-AIMR), Tohoku University, Katahira 2-1-1, Aoba-ku, Sendai, 980-8577, Japan

^*^Corresponding author. Email: yin.shu.b5@tohoku.ac.jp

**Experimental section**

**Reagents and chemicals.** Ce(NO_3_)_3_·6H_2_O, Fe(NO_3_)_3_·9H_2_O, Cr(NO_3_)_3_·9H_2_O, Benzoyl peroxide (BPO), and NaOH were obtained from Kanto Chemical Co., Inc. Co(NO_3_)_2_⋅6H_2_O, NaHCO_3_, Ni(NO_3_)_2_·6H_2_O, methyl methacrylate (MMA) and K_4_[Fe(CN)_6_]·3H_2_O were purchased from Fujifilm Wako Pure Chemical Corp. Carmine was procured from Tokyo Chemical Industry Co., Ltd. Tansei (Fe_2_O_3_) was supplied by Bijutsu Shuppan-Sha Co., Ltd.. All chemicals were used as received without further purification. All aqueous solutions were prepared using deionized (DI) water purified with a laboratory water purification system.

**Preparation of Cerium Carbonate^[1]^:** Under constant temperature conditions of 30-40 °C, a cerium nitrate solution was slowly added dropwise into 45 mL of a 0.33–0.45 M sodium bicarbonate solution and allowed to react for 24 hours. After completion of the reaction, the product was thoroughly washed with deionized water and dried to obtain flaky cerium carbonate.

**Preparation of Ce_2_(CO_3_)_3_·8H_2_O/FeOOH pearlescent pigments:** Two grams of the as-synthesized cerium carbonate were dispersed in 40 mL of deionized water under magnetic stirring to obtain a homogeneous white suspension. Subsequently, a predetermined molar amount of iron nitrate solution (see Table S1) and NaOH solution were simultaneously introduced into the dispersion, with the pH adjusted to 10. Under the alkaline synthesis conditions, the Ce_2_(CO_3_)_3_·8H_2_O substrate and FeOOH species carry opposite surface charges,[2-3] resulting in electrostatic attraction that facilitates the deposition of FeOOH onto the substrate surface. The mixture was further stirred for 30 min to ensure complete deposition of the iron species onto the cerium carbonate substrate. After the reaction, the suspension was allowed to stand for 12 h, thoroughly washed with deionized water, and freeze-dried to yield samples with different iron loadings, denoted as PF-L0–PF-L7 (detailed compositions are provided in Table S1).

**Preparation of CeO_2_/Fe_2_O_3_ Pearlescent Pigments:** The precursor samples (PF-L0–PF-L7) were calcined in a furnace at 800 °C for 2 h to obtain CeO_2_/Fe_2_O_3_ composite Multi-Biomimetic Optical Materials. The calcination temperature was determined based on TG-DTA analysis (Figure S4). The calcined products were then dispersed in absolute ethanol and ultrasonicated for 30 min to achieve uniform dispersion. Subsequently, the suspensions were thoroughly washed with deionized water and freeze-dried to yield the final Multi-Biomimetic Optical Materials. According to the different iron loading amounts, the obtained samples were denoted as PF-C0–PF-C7, with detailed parameters provided in the attached Table S1.

**Table S1.** Sample information of PF-L and PF-C

| Molar Fe Concentration (mol/g Substrate) | 0 | 1×10^-5^ | 5×10^-5^ | 1×10^-4^ | 3×10^-4^ | 5×10^-4^ | 1×10^-3^ | 2×10^-3^ |
| --- | --- | --- | --- | --- | --- | --- | --- | --- |
| Before sintering | PF-L0 | PF-L1 | PF-L2 | PF-L3 | PF-L4 | PF-L5 | PF-L6 | PF-L7 |
| After sintering | PF-C0 | PF-C1 | PF-C2 | PF-C3 | PF-C4 | PF-C5 | PF-C6 | PF-C7 |

**Preparation of Ce_2_(CO_3_)_3_·8H_2_O/Co, Ni, Cr-containing compounds pearlescent pigments:** Two grams of the as-synthesized cerium carbonate were dispersed in 50 mL of a 0.33-0.45 M sodium bicarbonate solution under magnetic stirring to obtain a homogeneous white suspension. Subsequently, a predetermined concentration of cobalt nitrate, nickel nitrate, or chromium nitrate solution was added dropwise to the pre-prepared cerium carbonate suspension. The mixture was further stirred for 30 min to ensure complete deposition of the compounds onto the substrate. After the reaction, the suspension was allowed to stand for 12 h, thoroughly washed with deionized water, and freeze-dried to yield samples with different loadings, denoted as PF-Co1–PF-Co7, PF-Ni1–PF-Ni7, and PF-Cr1–PF-Cr7. Detailed compositional ratios are provided in Table S2.

**Table S2.** Sample information of PF-Co, PF-Ni, and PF-Cr

| Molar Co/Ni/Cr Concentration (mol/g Substrate) | 1×10^-5^ | 5×10^-5^ | 1×10^-4^ | 3×10^-4^ | 5×10^-4^ | 1×10^-3^ | 2×10^-3^ |
| --- | --- | --- | --- | --- | --- | --- | --- |
| Co-containing compound | PF-Co1 | PF-Co2 | PF-Co3 | PF-Co4 | PF-Co5 | PF-Co6 | PF-Co7 |
| Ni-containing compound | PF-Ni1 | PF-Ni2 | PF-Ni3 | PF-Ni4 | PF-Ni5 | PF-Ni6 | PF-Ni7 |
| Cr-containing compound | PF-Cr1 | PF-Cr2 | PF-Cr3 | PF-Cr4 | PF-Cr5 | PF-Cr6 | PF-Cr7 |

**Preparation of Ce_2_(CO_3_)_3_·8H_2_O/Fe_2_O_3_ or Carmine pearlescent pigments:** Two grams of the as-synthesized cerium carbonate were dispersed in 40 mL of deionized water under magnetic stirring to obtain a homogeneous white suspension. Subsequently, a dispersion of Fe_2_O_3_ or carmine at a predetermined concentration was added to the cerium carbonate suspension. The mixture was further stirred for 30 min to ensure uniform deposition onto the substrate. After the reaction, the suspension was allowed to stand for 12 h, thoroughly washed with deionized water, and freeze-dried to yield samples with different loadings, denoted as PF-Fe1–PF-Fe5 and PF-CAR1–PF-CAR5. Detailed compositional ratios are provided in Table S3.

**Table S3.** Sample information of PF-Fe and PF-CAR

| Molar Fe_2_O_3_ Concentration (mol/g Substrate) | 1×10^-5^ | 5×10^-5^ | 1×10^-4^ | 3×10^-4^ | 5×10^-4^ |
| --- | --- | --- | --- | --- | --- |
| Fe_2_O_3_ | PF-Fe1 | PF-Fe2 | PF-Fe3 | PF-Fe4 | PF-Fe5 |
| Molar Carmine Concentration (mol/g Substrate) | 1×10^-6^ | 5×10^-6^ | 1×10^-5^ | 5×10^-5^ | 1×10^-4^ |
| Carmine | PF-CAR1 | PF-CAR2 | PF-CAR3 | PF-CAR4 | PF-CAR5 |

**Preparation of Ce_2_(CO_3_)_3_·8H_2_O/Fe_2_O_3_ or Carmine pearlescent pigments:** Two grams of the as-synthesized cerium carbonate were dispersed in 40 mL of deionized water under magnetic stirring to obtain a homogeneous white suspension. A K_4_[Fe(CN)_6_]·3H_2_O solution of predetermined concentration was prepared and mixed with a small amount of H_2_O_2_. Subsequently, an excess of Fe^3+^ ions was introduced into the solution to generate a blue Fe_4_[Fe(CN)_6_]_3_ precipitate.[4] The resulting precipitate was collected by filtration, thoroughly washed, and then re-dispersed into the cerium carbonate suspension. The mixture was further stirred for 30 min to ensure uniform deposition. After aging for 12 h, the suspension was washed with deionized water and freeze-dried to yield samples with different loadings, denoted as PF-CN1–PF-CN5. Detailed compositional ratios are provided in Table S4.

**Table S4.** Sample information of PF-CN

| Molar Fe_4_[Fe(CN)_6_]_3_ Concentration (mol/g Substrate) | 1×10^-6^ | 5×10^-6^ | 1×10^-5^ | 5×10^-5^ | 1×10^-4^ |
| --- | --- | --- | --- | --- | --- |
| Fe_4_[Fe(CN)_6_]_3_ | PF-CN1 | PF-CN2 | PF-CN3 | PF-CN4 | PF-CN5 |

**Preparation of colored PMMA:** BPO was dissolved in MMA in a glass flask. When the BPO was completely dissolved, the Multi-Biomimetic Optical Materials were added to the glass flask. Then, the mixture was magnetically stirred at 85℃ until the mixture closed glycerin. Finally, the mixture was transferred to a glass tube, cured at 50℃ for 24 hours, and reacted at 100℃ for 1 hour to obtain colored PMMA.

**Material characterizations**

X-ray diffraction (XRD, Bruker D2 Phaser) was employed at 30 kV and 10 mA with a Cu Kα (λ = 1.5418 Å) irradiation over a 2θ angle range from 10° to 50° to identify crystalline phases. Crystal structures were refined by the Rietveld method using the GSAS-EXPGUI software [5-6].

Scanning electron microscopy (SEM, JEOL JCM-5700) equipped with energy-dispersive X-ray spectroscopy (EDS, JED-272300 Series, JEOL) was employed to analyze the elemental composition.

Transmission electron microscopy (TEM) images and selected area electron diffraction (SAED) patterns were obtained using a JEM-2100F (JEOL) operated at an accelerating voltage of 200 kV. The instrument was equipped with an energy-dispersive X-ray spectroscopy (EDX) detector for elemental analysis. Scanning transmission electron microscopy (STEM) imaging and corresponding elemental mapping were also performed.

X-ray photoelectron spectroscopy (XPS, PHI5000 Versa Probe II) was applied to study the chemical composition and oxidation states, and the binding energy was calibrated by the C 1 s level at 284.6 eV.

UV-Vis-NIR diffuse reflectance spectroscopy (DRS) spectra were measured on a UV–vis spectrophotometer (V-670, JASCO) using Spectralon® as the white reflectance standard.

Color parameters in the CIE *L^*^a^*^b^*^* color space were recorded with a benchtop spectrometer (CR-10 Plus, Konica Minolta).

Optical micrographs were taken with an optical microscope (400-CAM106).

Thermogravimetry (TG) and differential thermal analysis (DTA) measurements were conducted using a TG-DTA 8122 instrument (Rigaku) in an air or nitrogen atmosphere.

Particle size distribution was investigated using a flow particle image analyzer (FPIA-3000S, SYSMEX).

The RGB (red, green, and blue) values were tested using ColorMeter 2.2.0 software.

Crystal structure models were generated using VESTA software.[7]

**Transfer matrix method (TMM)**

To quantitatively investigate the optical origin of the broadband reflection and its angular dependence, the reflectance spectra of the multilayer materials were calculated using the transfer matrix method (TMM). The multilayer structure was modeled as a one-dimensional dielectric stack consisting of alternating Ce_2_(CO_3_)_3_·8H_2_O (*n_Ce_* = 1.8)[8] and air gaps (*n_air_* = 1.0). The physical thickness of each layer, *d_m_*, was set according to the actual layer thickness measured from a single representative particle in the cross-sectional SEM image (Figure 1c).

The characteristic transfer matrix of the *m*-th dielectric layer is expressed as

$$M_{m}=\left[ \begin{matrix} cos\delta_{m} & i\frac{sin\delta_{m}}{\eta_{m}} \\ i\eta_{m}sin\delta_{m} & cos\delta_{m} \end{matrix} \right]$$

where *δ_m_* is the phase shift accumulated by the electromagnetic wave while propagating through the *m*-th layer:

$$\delta_{m}=\frac{2\pi}{\lambda}n_{m}d_{m}cos\theta_{m}$$

Here, *λ* is the wavelength in vacuum, *n_m_* and *d_m_* are the refractive index and physical thickness of the *m*-th layer, respectively, and *θ_m_* is the propagation angle within that layer. The refraction angle in each layer is determined according to Snell’s law:

$$n_{0}sin\theta_{0}=n_{1}sin\theta_{1}=\cdot\cdot\cdot=n_{m}sin\theta_{m}$$

The effective optical admittance *η_m_* depends on the polarization of the incident light and is given by

$$\eta_{m}=\left\{ \begin{aligned} n_{m}cos\theta_{m} TE \\ \frac{n_{m}}{cos\theta_{m}} TM \end{aligned} \right.$$

The total transfer matrix is obtained by sequentially multiplying the characteristic matrices of all layers:

$$M=\prod_{m=1}^{N} M_{m=}\left[ \begin{matrix} m_{11} & m_{12} \\ m_{21} & m_{22} \end{matrix} \right]$$

The complex amplitude reflection coefficient for each polarization is then calculated as

$$r=\frac{\left( m_{11}+m_{12}\eta_{s} \right)\eta_{0}-(m_{21}+m_{22}\eta_{s})}{\left( m_{11}+m_{12}\eta_{s} \right)\eta_{0}+(m_{21}+m_{22}\eta_{s})}$$

where *η_0_* and *η_s_* are the polarization-dependent optical admittances of the incident and exit media, respectively. The reflectance *R* is calculated using the following equation:

$$R=\left| r \right|^{2}$$

**Supplementary Note 1. Quantification of Fe-Containing Absorber Loading**

The phase compositions of the calcined samples were quantitatively analyzed by Rietveld refinement of the XRD patterns using the GSAS software. Because the FeOOH phase in the uncalcined samples was poorly crystalline or amorphous, reliable quantitative phase analysis could not be performed for these samples. In addition, the Fe_2_O_3_ contents in the calcined samples with relatively low Fe loadings were below the reliable quantification limit. Therefore, PF-C5 and PF-C6, which exhibited clearly distinguishable Fe_2_O_3_ diffraction peaks, were selected for quantitative refinement. The weight fractions of CeO_2_ and Fe_2_O_3_ were obtained from the refined phase compositions, and the corresponding Ce/Fe elemental ratios were subsequently calculated.

To quantitatively estimate the absorber loading, Rietveld refinement was performed on the XRD patterns of PF-C5 and PF-C6 (Figure S3a,b). PF-C5 contained 93.53 wt.% CeO_2_ and 6.47 wt.% Fe_2_O_3_, whereas PF-C6 contained 87.93 wt.% CeO_2_ and 12.06 wt.% Fe_2_O_3_ (Table S5). The corresponding Ce/Fe atomic ratios calculated from the refined phase fractions were 6.71:1 and 3.38:1, respectively, which were close to the theoretical values of 6.63:1 and 3.31:1 calculated from the precursor feeding amounts (Table S6). In addition, the post-reaction supernatants of PF-C5 and PF-C6 exhibited UV–vis spectra similar to that of water and showed no discernible Fe-related absorption in the visible region (Figure S3c), indicating a very low concentration of residual Fe species in solution. These results suggest that most of the introduced Fe species were deposited onto the substrate and that the absorber loading could be effectively controlled through the precursor feeding amount.

**Table S5.** Phase compositions of PF-C5 and PF-C6 determined by Rietveld refinement.

| Samples | CeO_2_ (wt.%) | Fe_2_O_3_ (wt.%) |
| --- | --- | --- |
| PF-C5 | 93.53 | 6.47 |
| PF-C6 | 87.93 | 12.06 |

**Table S6.** Comparison of the theoretical and refinement-derived Ce/Fe atomic ratios.

| Samples | Ce:Fe (Theoretical value) | Ce:Fe (Calculated value) |
| --- | --- | --- |
| PF-C5 | 6.63:1 | 6.71:1 |
| PF-C6 | 3.31:1 | 3.38:1 |

**Supplementary Note 2. Statistical Analysis of Layer Thickness**

The thicknesses of the inorganic layers and air gaps were measured from cross-sectional SEM images at multiple positions. The mean thickness ($\overline{x}$), sample standard deviation (*s*), and coefficient of variation (CV) were calculated according to the following equations:

$$\overline{x}=\frac{1}{n}\sum_{i=1}^{n} x_{i}$$

$$s=\sqrt{\frac{\sum_{i=1}^{n} {(x_{i}-\overline{x})}^{2}}{n-1}}$$

$$CV=\frac{s}{\overline{x}}\times100\%$$

where *x_i_* is the thickness of an individual inorganic layer or air gap and *n* is the total number of measurements.

The inorganic layers exhibited an average thickness of 329.59±314.03 nm with a coefficient of variation of 95.28%, while the air gaps showed an average thickness of 128.10±134.52 nm with a coefficient of variation of 105.01%. These high coefficients of variation quantitatively confirm the highly non-periodic and thickness-disordered nature of the multilayer structure.

**Supplemental Experimental Results**

**Table S7.** Values of atomic lattice parameters, positional, coordinates, occupancy and thermal parameters determined from Rietveld refinement of XRD pattern of Ce_2_(CO_3_)_3_·8H_2_O

| *α* (^°^) | *β* (^°^) | *γ* (^°^) | *a* (Å) | *b* (Å) | *c* (Å) | Volume (Å^3^) |
| --- | --- | --- | --- | --- | --- | --- |
| 90.0 | 90.0 | 90.0 | 9.52180(21) | 8.46368(12) | 8.91700(52) | 718.61(42) |
| Atom | Site | Occ | *x* | *y* | *z* | Uiso (Å^2^) |
| Ce1 | 2a | 1 | 0.00000 | 0.5024(7) | 0.4887(58) | 0.0555(16) |
| Ce2 | 2a | 1 | 0.00000 | 0.5545(6) | 0.0042(64) | 0.0890(23) |
| C1 | 2a | 1 | 0.00000 | 0.1882(20) | 0.0862(25) | 0.317(75) |
| C2 | 4b | 1 | 0.2574(13) | 0.4473(10) | 0.3183(11) | 0.112(38) |
| O1 | 2a | 1 | 0.00000 | 0.0432(20) | -0.0298(25) | 0.0456(15) |
| O2 | 4b | 1 | 0.0691(25) | 0.2753(25) | 0.0631(21) | 0.1857(16) |
| O3 | 4b | 1 | 0.2592(10) | 0.4254(9) | 0.4649(15) | 0.118(31) |
| O4 | 4b | 1 | 0.3797(10) | 0.4351(13) | 0.2550(10) | 0.0436(16) |
| O5 | 4b | 1 | 0.1508(9) | 0.4956(11) | 0.2595(13) | 0.0191(11) |
| O6  (H_2_O) | 4b | 1 | 0.331(11) | 0.2398(41) | 0.051(18) | 0.0614(13) |
| O7  (H_2_O) | 4b | 0.500 | 0.106(15) | 0.2643(64) | 0.647(20) | 0.0020(21) |
| O8  (H_2_O) | 4b | 0.500 | 0.170(19) | 0.1875(57) | 0.377(64) | 0.0109(22) |
| O9  (H_2_O) | 4b | 0.500 | 0.48(5) | 0.1639(116) | 0.292(30) | 0.0900(24) |
| O10  (H_2_O) | 4b | 0.500 | 0.444(23) | 0.1875(84) | 0.591(22) | 0.0156(16) |
| O11  (H_2_O) | 4b | 0.500 | 0.201(17) | 0.9544(62) | 0.95(7) | 0.0283(25) |
| O12  (H_2_O) | 4b | 0.500 | 0.148(15) | 0.0225(89) | 0.41(4) | 0.0344(21) |

**Table S8.** Selected interatomic distances (Å) of Ce_2_(CO_3_)_3_·8H_2_O

| Ce1_O3 | 2.5612(5)×2 |  |
| --- | --- | --- |
| Ce1_O4 | 2.6890(13)×2 |  |
| Ce1_O5 | 2.4983(10)×2 |  |
| Ce1_O6(H_2_O) | 2.76449(22)×2 |  |
| Ce1_O7(H_2_O) | 2.6630(5)×2 |  |
|  |  |  |
| Ce2_O2 | 2.50852(9)×2 |  |
| Ce2_O3 | 2.3263(5)×2 |  |
| Ce2_O4 | 2.5021(12)×2 |  |
| Ce2_O5 | 2.7367(11)×2 |  |
| Ce2_O9(H_2_O) | 3.0599(7)×2 |  |
| Ce2_O10(H_2_O) | 2.37531(16)×2 |  |
|  |  |  |
| C1_O1 | 1.6050(4) |  |
| C1_O2 | 1.00943(10)×2 |  |
|  |  |  |
| C2_O3 | 1.3199(8) |  |
| C2_O4 | 1.29793(28) |  |
| C2_O5 | 1.21400(24) |  |

**Figure S1.** (a) Photograph of pearls. Reproduced from Ref.[9] under CC BY 3.0. (Photograph by MASAYUKI KATO) (b) Microstructure of the pearl. Reproduced from Ref. [10] under CC BY 4.0. (c) Photograph of Steller's jay. Photograph by Nathean Peng, reproduced with permission from the author. (d) Feather microstructure of the Steller's jay.[11] Reproduced with permission. Copyright 2006, Company of Biologists. (e) Photograph of amelanotic Steller’s jay. Photograph from Project FeederWatch, Cornell Lab of Ornithology, reproduced with permission. (f) Feather microstructure of the amelanotic Steller’s jay.[11] Reproduced with permission. Copyright 2006, Company of Biologists.

**Figure S2.** (a) Simulated reflection spectra of the sample shown in Figure 1c at different incident angles. (b) Simulated reflection spectra of the samples shown in Figure 1c and Figure S5a–f. (c) Experimentally measured reflection spectrum of Ce_2_(CO_3_)_3_·8H_2_O. Simulations were performed using the transfer-matrix method, TMM.

The spectral overlap of these peaks (shown in Figure S2a) produces an overall broadband reflection response. In contrast to the simulated spectrum, which contains several distinguishable reflection features, the experimentally measured spectrum is relatively smooth over the visible region (shown in Figure S2c). We attribute this difference mainly to the different structural scales represented by the simulation and the experiment. The TMM model describes the multilayer structure of an individual representative particle, whereas the experimental spectrum is collected from a powder sample containing a large number of particles. Because the particles do not possess identical layer thicknesses, air-gap dimensions, or stacking configurations, their individual reflection features occur at slightly different wavelengths. Ensemble averaging over these structurally nonuniform particles therefore broadens and smooths the experimentally observed reflectance spectrum.

**Figure S3.** XRD Rietveld refinement of (a) PF-C5 and (b) PF-C6. (c) UV–vis absorption spectra of water, Fe(NO_3_)_3_ reference solutions with different concentrations, and the post-reaction supernatants collected during the preparation of PF-C5 and PF-C6.

**Figure S4.** TG-DTA trace of Ce_2_(CO_3_)_3_·8H_2_O and (b) Ce_2_(CO_3_)_3_·8H_2_O/FeOOH.

**Figure S5.** Side-view SEM image of Ce_2_(CO_3_)_3_·8H_2_O

**Figure S6.** Refined crystal structure of Ce_2_(CO_3_)_3_·8H_2_O.

**Figure S7.** Image-based particle size distribution (black bars) and cumulative size distribution (red curve) of (a) Ce_2_(CO_3_)_3_·8H_2_O/FeOOH and (b) CeO_2_/Fe_2_O_3_. Representative images of (c) Ce_2_(CO_3_)_3_·8H_2_O/FeOOH and (d) CeO_2_/Fe_2_O_3_ captured during the image-based particle size distribution analysis.

**Figure S8.** TEM image of Ce_2_(CO_3_)_3_·8H_2_O after prolonged exposure

**Figure S9.** XRD patterns of absorbing materials FeOOH without Ce_2_(CO_3_)_3_·8H_2_O substrate

**Figure S10.** SEM image and the corresponding EDS elemental mapping images of Ce_2_(CO_3_)_3_·8H_2_O (PF-L0)

**Figure S11.** SEM image and the corresponding EDS elemental mapping images of Ce_2_(CO_3_)_3_·8H_2_O/FeOOH (PF-L5)

**Figure S12.** Enlarged view of the side-view SEM image of CeO_2_ after sintering.

**Figure S13.** SEM image and the corresponding EDS elemental mapping images of CeO_2_ (PF-C0)

**Figure S14.** SEM image and the corresponding EDS elemental mapping images of CeO_2_/Fe_2_O_3_ (PF-C5)

**Figure S15.** (a-c) SEM image, (d) XRD, (e) Dynamic images and (f) Optical microscope image of CeO_2_. CeO_2_ obtained by calcination at 1000 °C.

**Figure S16.** XPS full scan spectrum of (a) Ce_2_(CO_3_)_3_·8H_2_O (PF-L0), (b) Ce_2_(CO_3_)_3_·8H_2_O/FeOOH (PF-L5), (c) CeO_2_ (PF-C0), and (d) CeO_2_/Fe_2_O_3_ (PF-C5)

**Figure S17.** Crystal structure diagrams of FeOOH and Fe_2_O_3_

**Figure S18.** Tanabe-Sugano (T-S) diagram for Fe^3+^ in octahedral coordination

**Figure S19.** Digital photographs of PF-L samples at different incident angles

**Figure S20.** Digital photographs of PF-S samples at different incident angles.

 **Figure S21.** Angle-resolved reflection spectra of (a) PF-L2, (b) PF-L4, (c) PF-L6, (a) PF-C2, (b) PF-C4 and (c) PF-C6

**Figure S22.** (a) Complementary color wheel.[12] (b) Absorption spectra of the absorbing materials. (c) Reflection spectra in the visible-light region of (c) PF-L, (d) PF-C, (e) PF-Fe, (f) PF-CAR, (g) PF-CN, (h) PF-Cr, (i) PF-Co and (j) PF-Ni.

**Figure S23.** XRD patterns of the (a) PF-Fe, (b) PF-CAR, (c) PF-CN, (d) PF-Cr, (e) PF-Co, and (f) PF-Ni series samples.

**Figure S24.** CIE *L^*^a^*^b^*^* color coordinates with photographs and CIE 1931 chromaticity coordinates of samples PF-Fe1–PF-Fe5. UV-VIS-NIR absorption spectra of Fe1-5 with dynamic images of the samples dispersed in water.

**Figure S25.** CIE *L^*^a^*^b^*^* color coordinates with photographs and CIE 1931 chromaticity coordinates of samples PF-CAR1–PF-CAR5. UV-VIS-NIR absorption spectra of Ca1-5 with dynamic images of the samples dispersed in water.

**Figure S26.** CIE *L^*^a^*^b^*^* color coordinates with photographs and CIE 1931 chromaticity coordinates of samples PF-CN1–PF-CN5. UV-VIS-NIR absorption spectra of CN1-5 with dynamic images of the samples dispersed in water.

**Figure S27.** CIE *L^*^a^*^b^*^* color coordinates with photographs and CIE 1931 chromaticity coordinates of samples PF-Cr1–PF-Cr7. UV-VIS-NIR absorption spectra of Cr1-7 with dynamic images of the samples dispersed in water.

**Figure S28.** CIE *L^*^a^*^b^*^* color coordinates with photographs and CIE 1931 chromaticity coordinates of samples PF-Co1–PF-Co7. UV-VIS-NIR absorption spectra of Co1-7 with dynamic images of the samples dispersed in water.

**Figure S29.** CIE *L^*^a^*^b^*^* color coordinates with photographs and CIE 1931 chromaticity coordinates of samples PF-Ni1–PF-Ni7. UV-VIS-NIR absorption spectra of Ni1-7 with dynamic images of the samples dispersed in water.

**Figure S30.** Digital photographs of PF-Fe samples at different incident angles

**Figure S31.** Digital photographs of PF-CAR samples at different incident angles

**Figure S32.** Digital photographs of PF-CN samples at different incident angles

**Figure S33.** Digital photographs of PF-Cr samples at different incident angles

**Figure S34.** Digital photographs of PF-Co samples at different incident angles

**Figure S35.** Digital photographs of PF-Ni samples at different incident angles

**Reference**

[1]	Yin, S., Minamidate, Y., Tonouchi, S., et al., "Solution synthesis of homogeneous plate-like multifunctional CeO_2_ particles," *RSC Advances* 2, no. 14 (2012).

[2]	Zhang, X., Yao, H., Lei, X., et al., "A comparative study for phosphate adsorption on amorphous FeOOH and goethite (α-FeOOH): An investigation of relationship between the surface chemistry and structure," *Environmental Research* 199(2021).

[3]	Hu, Y.-H., Xu, L., Li, M., et al., "Agglomeration behavior of petal-shaped cerium carbonate with different operating conditions," *Rare Metals* 37, no. 2 (2018).

[4]	Hornok, V., Dékány, I., "Synthesis and stabilization of Prussian blue nanoparticles and application for sensors," *Journal of colloid and interface science* 309, no. 1 (2007).

[5]	Larson, A., Von Dreele, R., "Gsas: General structure analysis system report LAUR 86-748," *Los Alamos National Laboratory, Los Alamos, NM* (1986).

[6]	Toby, B. H., "EXPGUI, a graphical user interface for GSAS," *Journal of applied crystallography* 34, no. 2 (2001).

[7]	Momma, K., Izumi, F., "VESTA 3 for three-dimensional visualization of crystal, volumetric and morphology data," *Applied Crystallography* 44, no. 6 (2011).

[8]	Nor Azman Shah, M. N., Abu Bakar, N. F., Shukri, A., "The precipitation of large cerium carbonate by Na₂CO₃ in various condition and its characterization," (2018).

[9]	Kato, M. 2011, "Akoya pearl." Wikimedia Commons.

[10]	Ozaki, R., Kikumoto, K., Takagaki, M., Kadowaki, K., Odawara, K., "Structural colors of pearls," *Scientific Reports* 11, no. 1 (2021).

[11]	Shawkey, M. D., Hill, G. E., "Significance of a basal melanin layer to production of non-iridescent structural plumage color: evidence from an amelanotic Steller's jay (Cyanocitta stelleri)," *Journal of Experimental Biology* 209, no. 7 (2006).

[12]	Chekkaramkodi, D., Ahmed, I., Jacob, L., Butt, H., "3D printed UV-sensing optical fiber probes: manufacturing, properties, and performance," *Scientific Reports* 14, no. 1 (2024).
